# Evaluating community engagement in global health research: the need for metrics

**DOI:** 10.1186/s12910-015-0033-9

**Published:** 2015-07-01

**Authors:** Kathleen M. MacQueen, Anant Bhan, Janet Frohlich, Jessica Holzer, Jeremy Sugarman, the Ethics Working Group of the HIV Prevention Trials Network

**Affiliations:** Social and Behavioral Health Sciences, FHI 360, 359 Blackwell Street, Durham, NC 27514 USA; Bioethics and Global Health, Bhopal/Pune, India; Vulindlela Clinical Research Site, CAPRISA, University of KwaZulu-Natal, Durban, South Africa; Hofstra University, Hempstead, NY USA; Berman Institute of Bioethics, Johns Hopkins University, Baltimore, MD USA

**Keywords:** Research ethics, Community engagement, Participatory research, Global health, Evaluation, Metrics

## Abstract

**Background:**

Community engagement in research has gained momentum as an approach to improving research, to helping ensure that community concerns are taken into account, and to informing ethical decision-making when research is conducted in contexts of vulnerability. However, guidelines and scholarship regarding community engagement are arguably unsettled, making it difficult to implement and evaluate.

**Discussion:**

We describe normative guidelines on community engagement that have been offered by national and international bodies in the context of HIV-related research, which set the stage for similar work in other health related research. Next, we review the scholarly literature regarding community engagement, outlining the diverse ethical goals ascribed to it. We then discuss practical guidelines that have been issued regarding community engagement. There is a lack of consensus regarding the ethical goals and approaches for community engagement, and an associated lack of indicators and metrics for evaluating success in achieving stated goals. To address these gaps we outline a framework for developing indicators for evaluating the contribution of community engagement to ethical goals in health research.

**Summary:**

There is a critical need to enhance efforts in evaluating community engagement to ensure that the work on the ground reflects the intentions expressed in the guidelines, and to investigate the contribution of specific community engagement practices for making research responsive to community needs and concerns. Evaluation mechanisms should be built into community engagement practices to guide best practices in community engagement and their replication across diverse health research settings.

## Background

Community engagement in research has gained momentum as an approach to improving research, to helping ensure that community concerns are taken into account, and to informing ethical decision-making when research is conducted in contexts of vulnerability [1–3]. The term *community* generally denotes a group of people with some kind of shared social identity, while the term *engagement* indicates an interactive relationship between a community and a research entity. The phrase *community engagement* has been used to describe a wide range of practices.

National and international bodies now recognize the importance of community engagement in research. For example, the Wellcome Trust [4] and the Bill and Melinda Gates Foundation [5, 6] have provided funding to examine the ethical aspects of community engagement in international research. The US National Institutes of Health (NIH) has supported community-engaged research through its Clinical and Translational Science Award (CTSA) program and the NIH Division of AIDS (DAIDS) provides core funding to support community engagement via its research networks [7]. Further, major international research efforts such as H3Africa have embedded community engagement efforts [8]. A review of international ethics guidance documents published in 2004 identified community engagement to be a central guiding principle [9]. Interestingly, a review published by the same group four years earlier, which did not have developing countries as an explicit focus, did not include community engagement as a guiding principle [10], suggesting a view that community engagement was essential in particular settings and/or a rather rapid emergence of global consensus on its importance.

Despite such broad support, guidelines and scholarship regarding community engagement are arguably unsettled [11], which can make it difficult to implement and assess, potentially resulting in missed opportunities, wasted resources and poor decisions. In this paper we first describe normative guidelines on community engagement that have been offered by national and international bodies in the context of HIV-related research, which set the stage for similar work in other health related research. Next, we review the scholarly literature regarding community engagement, outlining the diverse ethical goals ascribed to it. We then discuss practical guidelines that have been issued regarding community engagement, the lack of consensus regarding their ethical goals and approaches, and the associated lack of indicators and clear metrics for evaluating success in achieving such goals. We then offer a framework for developing indicators as a critical step toward evaluating community engagement in health research.

## Discussion

### Guidelines on community engagement

#### Statements of principle in HIV-related research

Since early in the AIDS pandemic, substantial attention has focused on community engagement in HIV related research, with several national and international bodies offering particular guidelines for this purpose. Following an extensive two-year global consultation process UNAIDS in 2000 put forward a comprehensive set of guidelines for the implementation of HIV vaccine trials that referenced community participation. Guidance Point 5 stated that “community representatives should be involved in an early and sustained manner in the design, development, implementation, and distribution of results of HIV vaccine research” that included establishment of “a continuing forum for communication and problem-solving”[12](p. 19). In the updated and expanded 2007 *Ethical Considerations in Biomedical HIV Prevention Trials*, the language was refined and the need for community engagement received increased emphasis:

Guidance Point 2: Community Participation

*To ensure the ethical and scientific quality and outcome of proposed research, its relevance to the affected community, and its acceptance by the affected community, researchers and trial sponsors should consult communities through a transparent and meaningful participatory process which involves them in an early and sustained manner in the design, development, implementation, monitoring, and distribution of results of biomedical HIV prevention trials* [13].

Also in 2007, UNAIDS and AVAC (a global advocacy organization for HIV prevention research) jointly authored the *Good Participatory Practice Guidelines for Biomedical HIV Prevention Trials* (GPP-HIV). Revised in 2011, GPP-HIV includes guiding principles for implementation of community and stakeholder engagement [14]. Subsequently, the Stakeholder and Community Engagement Workgroup of the Critical Path to TB Drug Regimens collaborated with AVAC to adapt GPP-HIV for tuberculosis (TB) research, releasing the Good Participatory Practice Guidelines for TB Drug Trials (GPP-TB) in 2012 [15, 16]. The importance of community engagement as a cross-cutting ethical issue for HIV, TB and malaria vaccine trials was underscored in a report from a 2009 consultation sponsored by the Ethics, Law and Human Rights Collaborating Centre of the WHO/UNAIDS African AIDS Vaccine Programme [17].

In 2009 the HIV Prevention Trials Network’s (HPTN) ethics guidance for research [18], directly addressed community engagement as an ethical obligation in guidance point 3:

*In order to ensure that HPTN research is appropriate as well as scientifically and ethically sound, relevant communities will be engaged in a meaningful process that will help guide the research from protocol development to dissemination of results.*

Guidance point 4, framed as aspirational, separately addressed local capacity-building and establishing partnerships within local communities.

Community engagement has been explicitly incorporated into some national guidelines for clinical research broadly and HIV research specifically. For example, South Africa’s National Health Research Ethics Council’s *Guidelines for Good Practice in the Conduct of Clinical Trials with Human Participants in South Africa* recommended that research ethics committees require investigators to provide plans to consult with community representatives as well as involve communities during the research and in disseminating research findings from the study. The guidelines note the importance of involving communities in research when they are deemed “vulnerable” and explicitly require community involvement in the design and conduct of population-focused HIV prevention research [19]. The Guidelines further recommend that sponsors establish community advisory groups (CAGs) for research carried out at a community level (e.g., vaccine trials) as a means to “ensure adequate consultation with civil organisations that may exist within affected communities at all phases of the trial” [19](page 29).

#### Scholarly discourse

In tandem with the promulgation of ethics guidelines, a rich discussion about the ethical goals of community engagement can be found in the scholarly literature, as outlined in Table 1.

**Table 1:** Ethical goals of community engagement (listed chronologically by publication date)

Weijer and Emanuel, 2000 [20]
Protect communities in research
Emanuel et al., 2004 [9]
Minimize the possibility of exploitation
Increase the likelihood that the research will have a long-term impact
Demonstrate an awareness of and respect for cultural differences between the researchers, sponsors and communities
Respect for recruited participants and study populations
Dickert and Sugarman, 2005 [21]
Enhance protections for communities
Enhance benefits for communities
Enhance legitimacy for the research
Partners share responsibility for the conduct of the research
Nuffield Council of Bioethics, 2005 [53]
Enhance respect for communities
Tailor research to better meet the needs of communities
Tindana et al., 2007 [6]
Ensure the relevance of research
Assess whether relevant research is culturally and practically acceptable in the context it is intended
Ensure that community disruption is minimized, i.e., avoiding the displacement of local medical staff from pressing local needs
Avoid exploitation, by ensuring a fair distribution of the benefits of research
Take into account the ethical hazards that may be part of the social, economic, and political landscape of the community
Ahmed and Palermo, 2010 (COPR) [1]
Communities and investigators share power and responsibility equitably
Diverse perspectives and populations are included in an equitable manner
The research project results in mutual benefit for all partners
All partners receive equal respect
UNAIDS and AVAC, 2011 [14]
Ensure the ethical and scientific quality and outcome of proposed research
Ensure relevance of research to the affected community
Ensure acceptance of research by the affected community
Participants in the Community Engagement Consent Workshop, Kilifi Kenya [11]
Support research that is respectful to individuals and communities where social value is maximized
Critical Path to TB Drug Regimens, 2012 [15]
Ensure that disparities and inequalities are not inadvertently replicated or reinforced
Ensure that power dynamics do not disadvantage some stakeholders more than others (minimize the threat of exploitation)
Ensure that the burden associated with TB drug trials is fully apprehended and protocols are adjusted to minimize the burden
Prioritize the management of stigma and involuntary isolation
Ensure emerging challenges are addressed in the new era of TB drug trials
King, et al., 2014 [24]
Identifying and managing non-obvious risks and benefits
Expanding respect beyond the individual to the stakeholder community
Building legitimacy for the research project

Building on a history of community engagement in public health practice and growing attention to community engagement in research, in 2000, Weijer and Emanuel argued that the ethical goal of community engagement in research was to protect communities [20]. They recommended a variety of processes by which such protection could be realized, largely based on the characteristics of the community or communities involved in the research in question. In 2004, Emanuel and co-authors listed “collaborative partnership” as one of the eight criteria for ethical research in developing country settings. They argued that collaborative partnership with the community minimizes the possibility of exploitation, increases the likelihood that the research will have a long-term impact, and demonstrates an awareness of and respect for cultural differences between the researchers, sponsors and communities [9]. Another criterion listed was “respect for recruited participants and study populations,” which includes, among other things, the requirement that researchers inform participants and the community if new information arises during the course of the research and develop strategies to inform communities of the results of the research.

Though community consultation may be conceived of as a narrow form of community engagement in research, the underlying motivations for undertaking each are similar if not the same [6]. In 2005, Dickert and Sugarman articulated ethical goals for community consultation including enhanced protection, enhanced benefits, legitimacy, and shared responsibility for the conduct of the research [21].

In 2007 Tindana and co-authors [6] summarized the goals of community engagement from a number of sources including the National Bioethics Advisory Commission [2], the Nuffield Council on Bioethics [22], and the Council for International Organizations of Medical Sciences [23]. In general, they found that the ethical goals of community engagement were described in terms of a concern for the well-being of communities and protection of their interests, including fair representation of communities in projects; attention to equitable participation in partnerships and the research process; fair distribution of the benefits of research; and shared responsibility for the conduct of the research. They also noted that the extant guidance generally indicated why, from an ethical perspective, communities should be engaged in research: for their benefit, to reduce harms, and to result in more appropriate research. More recently, King and colleagues have proposed a universal framework that emphasizes the “human infrastructure” of relationships between researchers and communities and aligns community engagement goals with three core ethical responsibilities: (1) identifying and managing non-obvious risks and benefits; (2) expanding respect beyond the individual to the stakeholder community; and (3) building legitimacy for the research project [24].

A Community Engagement and Consent Workshop held in Kilifi, Kenya, in 2011 brought together groups engaged in research and practice on community engagement and informed consent from across the globe and with a wide diversity of disciplinary backgrounds. The workshop resulted in a critical examination of the characterization, conduct, and evaluation of community engagement and consent processes in diverse health research contexts [11]. This examination highlighted the lack of clarity in the range and scope of goals claimed for community engagement, noting that they “can be broadly divided into those that are more instrumental, such as engaging communities to improve the quality of research (or simply satisfying funders), and those that are more intrinsic such as engaging communities to show respect or to ensure a sense of inclusion” [11] (p. 9). Workshop participants identified a range of interrelated reasons for greater clarity of community engagement goals in research, including potential tensions between differing aims, the potential for negative impacts including unintended perverse outcomes, and the limits to the ethical issues that community engagement “can resolve in research, including those related to historical and background injustices and inequities and poorly resourced health systems” (p. 10). They highlighted the importance of considering goals before and throughout studies to support planning and evaluation of community engagement activities.

#### Practical guidance

In parallel with the scholarly literature regarding community engagement, those engaged in research have articulated practical guidance. Early examples tended to focus heavily on Community Advisory Boards (CABs) as a mechanism to address ethical challenges and minimize lapses related to, for example, meeting informed consent requirements and contextual meanings of risks and benefits [25–27]. However, the limitations of CABs have also been increasingly noted including challenges in assuring appropriate representation, mitigating power imbalances, and balancing CAB independence with research-based support [28]. CABs are now commonly complemented by and balanced with other forms of engagement and participation to bring a diversity of community voices, perspectives and concerns to the forefront including the use of traditional community assemblies [29], qualitative research [30–33], and deliberative engagement processes [34].

In 2009, the National Institute of Allergy and Infectious Diseases (NIAID), NIH, published a report, *Recommendations for Community Involvement in National Institute of Allergy and Infectious Diseases HIV/AIDS Clinical Trials Research*. The report described principles of community engagement, adapted from a 1997 report of the United States Centers for Disease Control and Prevention (CDC) and Agency for Toxic Substances and Disease Registry (ATSDR) Committee on Community Engagement [35]. The NIAID recommendations apply particularly to CABs, indicating that CAB members are expected to provide community input into the research and foster a partnership between researchers and the communities in which and with whom the research is being conducted [3]. The recommendations delineate the different roles and responsibilities of CAB members at different levels of the research enterprise including the international research network level as well as the local research site. The 1997 CDC/ATSDR guidelines were extensively updated in 2011 in collaboration with the Clinical and Translational Science Awards Consortium [36]; and in 2014 an updated version of the NIAID report was developed by Community Partners, a global group of community representatives affiliated with the HIV/AIDS clinical trials networks funded by the National Institutes of Health (NIH), in collaboration with NIAID [37].

The NIH’s Council of Public Representatives (COPR), which provides advice to the Director of the NIH, in 2010 developed frameworks for community engagement in research education (including research values, strategies to operationalize each value, and potential outcomes of their use and peer review) and guidance for peer-review of such work [1]. COPR called for application of the values and frameworks within the US but noted that the distinction between international and domestic work, and work with communities in developed and developing nations, may be unnecessary when considering the principles and guidelines for community engagement.

Lavery and colleagues offered a set of guidelines in 2010 derived from their empirical work on community engagement [38]. They outlined a set of ‘points to consider’ that provide practical guidance on community engagement in international research. These may be viewed as steps on the path to achieving ethical goals via community engagement, for example, characterizing and building knowledge of communities; ensuring the purpose and goals of the research are clear to the community; identifying, mobilizing and developing relevant attitudes about the proposed research; and ensuring adequate opportunities and respect for dissenting opinions.

In 2014 the Human Heredity and Health (H3Africa) Consortium posted *Guidelines for Community Engagement* in support of their mission to foster genomic research expertise in Africa as a tool for addressing health inequities. The guidelines identified community engagement goals focused on securing the support of the community for a research project, improving understanding of the research process, and soliciting views and inputs of community members on aspects of the research [39] (p. 9).

### From ethical goals to evaluation of practice

An oft-cited concern with community engagement in research is the difficulty in assessing the effectiveness of community engagement in general, or of practices considered to be community engagement in particular [40]. In order to assess effectiveness, however, the desired goal needs to be clearly defined since it provides a logical foundation for developing appropriate indicators of success. Thus, evaluation of community engagement is challenged by the diversity of ethical goals attributed to it even among closely related guidance documents. For example, while there is considerable overlap between the Good Participatory Practices document first developed by UNAIDS/AVAC for biomedical HIV prevention research (GPP-HIV) and subsequently adapted by CPTR for TB clinical trials research (GPP-TB), they differ with regard to the framing of ethical goals (Table 1). It can be argued that a diversity of ethical goals is needed in order for community engagement to be responsive to the contextual nuances of both the research and the setting where it takes place. Consequently, this would place obvious limitations on the generalizability of any given evaluation design. It could also be argued that a core set of generic ethical goals exist across all community engagement contexts for research [24].

A further limitation in the literature and in existing guidelines is the use of community engagement to describe highly varied practices, for which different ethical standards may exist. This stems in part from the use of “community” to describe a broad range of groupings of individuals, from local communities to disease communities to broader social groupings, such as racial or ethnic groups and society as a whole [1, 21, 20]. In this regard, the GPP-HIV and GPP-TB documents are noteworthy in that they explicitly address the role of stakeholders at multiple levels from the local to the global. Stakeholders may coalesce into a variety of communities, some of which overlap and others that are highly distinct.

In order to understand how well community engagement in research is working, standard and reliable measures are needed to gauge its success. Some measures have been developed for community engagement broadly, and for community-based participatory research in particular, though they are few and the evidence generated to date is thin [41, 42]. Most often, evaluation focuses on the process of engagement and the contribution of engagement to successful research implementation; no widely available resources have yet been developed to explicitly evaluate how and whether specific community engagement practices lead to enhanced ethical outcomes in research.

Some instructive efforts are underway to evaluate the impact of community engagement on research practice. A study that used surveys to assess the acceptability of exception from informed consent for research in emergency settings found that interactive community consultations resulted in significantly higher acceptability and higher level recall of study content than non-interactive consultations [43]. Approaches to evaluating community engagement activities were briefly described in the H3Africa guidelines including use of theory-based methodology, monitoring participation at events and meetings, documentation to track discussions, and use of interviews, focus groups and surveys [39]. An example informing the H3Africa guidelines was the use of qualitative research to evaluate the informed consent process, including a community engagement component, for malaria-related genetics research in Ghana [44]. More recently, the first author of this paper (MacQueen) is leading a project to develop and pilot an evaluation framework for GPP-TB [45]. The data emerging from these and similar efforts will help inform deliberations about methods of evaluation and identify effective, ineffective, and potentially detrimental or harmful methods of engagement.

As a step toward developing an evaluation framework specific to the ethical goals of community engagement, Table 2 categorizes the ethical goals of community engagement in research that are depicted in Table 1. These ethical goals provide a starting point for asking what would constitute evidence that community engagement practices contribute to the achievement of a particular goal. Potential indicators reflective of the kind of evidence that would need to be generated to show that particular community engagement practices contributed to achieving particular ethical goals are then identified. Ethical goal statements such as those included in Table 2 could be used to develop more detailed logic models commonly used in public health to describe strategies, inputs, outcomes, and impacts [46, 47]. The logic models, in turn, would support systematic evaluation of the contribution of community engagement practices to the broad range of ethical claims being made. Such an approach would help to strengthen the effectiveness of practice, identify areas in need of strengthening, and ensure that resources are directed toward activities that enhance achievement of clearly articulated ethical goals. Conversely, if specific ethical goals cannot be achieved via community engagement in practice, conceptual work regarding such goals should be reconsidered in light of the emerging data.

**Table 2:** Potential indicators for evaluating the contribution of community engagement (CE) to ethical goals

Ethical goal	Potential indicators
Broadly protect communities in research	• Procedures developed through CE exist to investigate events that have been reported indirectly, such as through a third party, taking account of confidentiality issues [14]
Weijer and Emanuel, 2000 [20]; Dickert and Sugarman, 2005 [21]; King et al., 2014 [24]	• Procedures developed through CE exist for reporting social harms, and whether these are to be reported to sponsors, ethics committees, and regulatory bodies if not specifically required by them [14]
	• Documentation that stakeholders reflective of the potential reach of the research are identified and actively engaged, beyond individual research participants [24]
Minimize the possibility of exploitation	• Procedures developed through CE exist to ensure community members know where the research is being conducted and by whom [3]
Emanuel et al., 2004 [9]; Tindana et al., 2007 [6]; Critical Path to TB Drug Regimens, 2012 [15]	• Documentation of use of appropriate mechanisms to ensure community members understand research concepts (e.g., the difference between research and clinical care) [3]
Increase the likelihood that the research will generate fair benefits locally	• Evidence that CE empowered stakeholders to develop systems that are useful to the community, build local capacity and gain control over their lives [54]
Emanuel et al., 2004 [9]; Dickert and Sugarman, 2005 [21]; Nuffield Council of Bioethics, 2005 [53]; Ahmed and Palermo, 2010 (COPR) [1]; UNAIDS and AVAC, 2011 [14]	• Evidence that community members used the knowledge gained through CE to improve community members’ health and well-being [1]
	• Research benefits identified during CE are demonstrated to accrue to research participants and participant communities [1]
Ensure awareness of and respect for cultural differences	• Evidence that researchers and research staff are aware of cultural differences relevant to the research and have established procedures that respect those differences and allow for them in research [24]
Emanuel et al., 2004 [9]; Tindana et al., 2007 [6]; King et al., 2014 [24]	• Evidence that community members feel the research procedures and process was/is respectful to their culture [24]
	• Evidence of on-going relationships and open-ended discussions with key stakeholders regarding the research and whether it is respectful of cultural differences [24]
Ensure respect for recruited participants and study populations	• Evidence that trust between communities and investigators increases following implementation of CE [1]
Emanuel et al., 2004 [9]; Nuffield Council of Bioethics, 2005 [53]; Ahmed and Palermo, 2010 (COPR) [1]; King et al., 2014 [24]	• Evidence of listening to, acknowledging, and being responsive to stakeholders [24]
Legitimacy of the engagement process	• Documentation of who in a community is engaged in deliberation and discussion about the research and the extent to which they represent the views of the larger community and relevant minority groups within communities [24]
Dickert and Sugarman, 2005 [21]; UNAIDS and AVAC, 2011 [14]; King et al., 2014 [24]	• Processes are in place to air disagreements and discuss the concerns and interests of the stakeholder community [24]
	• Documentation of clearly articulated goals for CE and tools for tracking progress in achieving those goals [24]
Partners share responsibility for the conduct of the research	• CAB provides documented feedback on the protocol, consent materials and/or recruitment materials [3]
Dickert and Sugarman, 2005 [21]; Ahmed and Palermo, 2010 (COPR) [1]	• Documentation that community members share suggestions for research with researchers or are comfortable with the proposed approach [3]
	• Documentation that researchers and staff respond to community input [3]
	• Documentation that communities participate in research throughout the entire process, including determining the importance of the problem, assessing the value of the research and conducting the study [54, 9]
	• Documentation of substantive community contributions to the design and evaluation of the informed consent process [54]
	• Documentation of substantive CAB member participation on protocol development teams and scientific committees [3]
	• Procedures are in place to actively probe participants and encourage reporting of social harms [14]
	• Documentation that CAB meeting(s) are held with community stakeholders to discuss study design, eligibility, and implementation [3]
	• Documentation that CAB meetings are held with researchers and research staff to discuss research results and that the wider community is informed of research results [3, 9]
Minimize community disruption	• Evidence that conflicts, misunderstandings, and criticisms are minimized or prevented through CE [1]
Tindana et al., 2007 [6]	
Ensure that disparities, inequalities and stigma are not inadvertently replicated or reinforced	• Evidence of changed norms and behaviors around disease-related stigma in the community due to CE [54]
Tindana et al., 2007 [6]; Critical Path to TB Drug Regimens, 2012 [15]	• Evidence that traditionally underserved communities increase their power as a result of CE [1]
	• Evidence that financial and other rewards of research identified through CE are shared fairly [9]

While Table 2 points toward the development of an evaluation framework for community engagement, the next steps must be done with considerable care. For example, it is challenging to reconcile the inherent complexity of the multiple layers of social embeddedness described in the GPP documents with the requirements of most evaluation models. Community engagement is meant to be a dynamic process that is imbued with feedback loops that result in adaptive change by stakeholders and transformation of the relationship context. Such challenges can be seen in a post-hoc evaluation of the community engagement program of the FEM-PrEP pre-exposure prophylaxis trial for HIV prevention among African women, which situated and assessed activities in the context of the 2011 GPP-HIV guidelines [48]. A process evaluation of community engagement for a paediatric randomized controlled malaria vaccine trial at three sites in Kilifi, Kenya further illustrates the need for evaluation aligned with iterative and evolving interactions among all stakeholders inclusive of researchers [49]. To be useful and informative, evaluation must move beyond a simple cause-effect framing of “does community engagement work?”

Two evaluation approaches appear to be promising in this regard. The *theory of change* approach makes explicit the presumed pathways by which a program may lead to desired goals [50, 51]. This in turn helps in identifying indicators, causes, and outcomes to include in the evaluation as well as appropriate methods to determine if the presumed pathways hold up as theorized. The *realist evaluation* approach takes a somewhat different tack by asking “what works for whom in what circumstances and in what respects, and how?” [51] Here the emphasis is on elucidating the relationships among context, change mechanisms, and outcomes to identify factors that may affect both the intervention and its outcome. Realist evaluation presumes repeated iterations between theory development and empirical investigation at the micro level [52]. Theory of change and realist evaluation are potentially complementary approaches that show promise but are also relatively new and have weaknesses with regard to methodological clarity [51, 50]. A richer exploration of the application of logic models, theory of change, and realistic evaluation to the ethical aspects of community engagement is needed.

## Summary

There is a critical need to enhance work in evaluating community engagement—to ensure that the work on the ground reflects the intentions expressed in the guidelines, and also to investigate the contribution of specific community engagement practices for making research responsive to community needs and concerns. We encourage further research in this area, and recommend that research groups nest evaluation mechanisms in their community engagement practices to be able to develop a refined and evidence-based understanding of what aspects of current community engagement work is effective, and to identify areas where further work is needed. Evaluation designs should reflect explicit statements about the goals of the community engagement work undertaken. This will also help in evolving a set of best practices in community engagement that can be replicated across various settings. A coherent set of community engagement goals and indicators would also assist research ethics committees in deciding the minimal community engagement practices required of those applying for ethics review and approval.
